# Myxedema Ascites: A Rare Presentation of Uncontrolled Hypothyroidism

**DOI:** 10.7759/cureus.912

**Published:** 2016-12-05

**Authors:** Sameen Khalid, FNU Asad-Ur-Rahman, Aamer Abbass, Dwayne Gordon, Khalid Abusaada

**Affiliations:** 1 Internal Medicine Residency, Florida Hospital-Orlando; 2 Graduate Medical Education, Florida Hospital-Orlando

**Keywords:** myxedema, ascites, hypothyroidism

## Abstract

Less than four percent of patients with hypothyroidism develop ascites. Ascites as the presenting feature of hypothyroidism is uncommon, hence diagnosis is often delayed. Once it is diagnosed, treatment of hypothyroidism leads to quick clinical improvement in ascites. We report a case of a female patient who presented with ascites secondary to severe hypothyroidism and discuss the diagnostic characteristics of the ascitic fluid in myxedema ascites on the basis of literature review.

## Introduction

Primary hypothyroidism is a common clinical condition that can manifest in different ways. Typical symptoms include cold intolerance, weight gain, constipation, menstrual abnormalities, slow mentation, and fatigue. Severe, uncontrolled hypothyroidism can lead to accumulation of fluid in body cavities, presenting as pericardial effusion, pleural effusion and ascites. Ascites caused by hypothyroidism is rare, occurring in less than four percent of hypothyroid patients [1-5]. Hypothyroidism is also an uncommon cause of ascites and is seen in only one to five percent of patients with ascites [3]. Ascites as the only feature of hypothyroidism is not common, hence diagnosis is often delayed. Treatment of underlying hypothyroidism leads to quick clinical improvement [4-6]. We report a case of a female patient who presented with ascites secondary to severe hypothyroidism and discuss the diagnostic characteristics of the ascitic fluid in myxedema ascites on the basis of literature review. Informed consent was obtained from the patient for this study.

## Case presentation

A 56-year-old Caucasian female presented with progressive painless abdominal distension associated with bloating and constipation for four weeks. She was diagnosed with Hashimoto's thyroiditis thirty years ago; however, she stopped taking thyroxine supplementation six months prior to presentation. She denied any alcohol consumption or history of known hepatitis B or C. An examination revealed normal vital signs, mild periorbital edema, and dry pale skin. The thyroid gland was not palpable. A cardiopulmonary examination did not show S3 gallop, jugular venous distension, or other features suggestive of heart failure. Her abdomen was distended without appreciable visceromegaly. There was fluid thrill and shifting dullness along with hypoactive bowel sounds. No palmar erythema, asterixis, jaundice, or spider angiomata were appreciated. A lower extremity examination showed mild, non-pitting edema extending halfway up the shin.

The initial workup revealed hemoglobin of 10.7 g/dl with macrocytosis (mean corpuscular volume of 103 fl), platelet count of 204 x 103/µL and white blood cell (WBC) count of 3.2 x 103/µL with normal differentials. The complete metabolic panel was significant for mildly elevated aspartate aminotransferase (AST; 123 units/L) and alanine aminotransferase (ALT; 75 units/L). The international normalized ratio was 0.89. Creatine kinase (CK) was mildly elevated (839 units/L). The lipid panel revealed elevated triglycerides (178 mg/dl), total cholesterol (224 mg/dl) and low-density lipoprotein (LDL; 128 mg/dl). The thyroid function tests demonstrated a profoundly elevated thyroid stimulating hormone level (TSH; >100 units/ml) (normal range: 0.4-4.5 units/ml) with very low level of free T4 (0.2 ng/dl) (normal range: 0.58-1.64 ng/dl).

One month prior to this presentation, she was hospitalized in another institution for similar complaints of weight gain and new onset ascites. During that hospitalization, she underwent diagnostic paracentesis to evaluate the ascites. Ascitic fluid was found to be an exudate with a high protein content of 3.5 g/dl and serum ascites albumin gradient (SAAG) of 0.9. The patient underwent a second diagnostic and therapeutic ascitic tap at our facility that revealed exudative fluid with elevated total protein of 3.9 g/dl, an albumin level of 2.9 g/dl with a SAAG of 1.0. The total WBC count was 497/µL with two percent neutrophils, 33% lymphocytes, two percent monocytes and 50% macrophages. Glucose was 92 mg/dl, triglycerides were 49 mg/dl and lactate dehydrogenase was 174 units/L. Gram stain and cytology were negative. The bacterial, fungal, and mycobacterial cultures were also negative.

**Figure 1:**
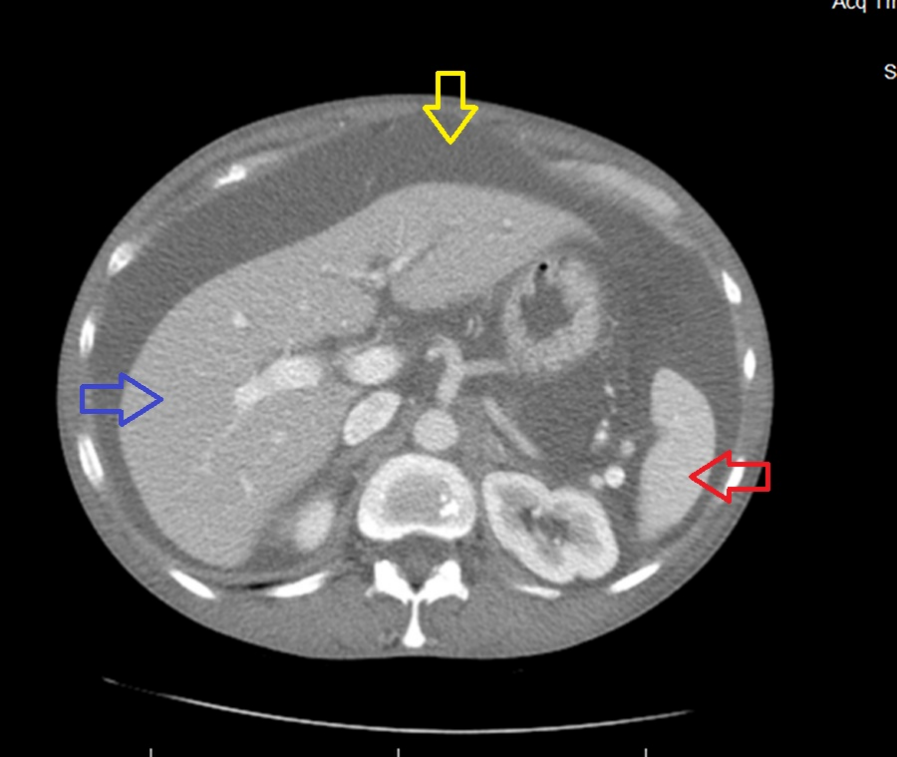
CT of the abdomen showing ascites (yellow arrow), normal sized liver (blue arrow) and spleen (red arrow)

An extensive workup had been done at the other hospital to elucidate the underlying etiology of ascites. The hepatitis panel was negative. The tumor markers (CA 19-9 and CA 125) were negative. A Doppler ultrasound did not show any abnormality in hepatic or portal vasculature. The ultrasound and computed tomography (CT) of the abdomen and pelvis revealed massive ascites and a normal sized liver and spleen, as can be seen in Figure 1. The ovaries were not enlarged and no pelvic masses were seen.

The CT of the abdomen revealed prominence of the pancreatic head relative to its body and tail, without pancreatic duct dilatation. A magnetic resonance imaging (MRI) of the abdomen revealed pancreatic head enlargement as well. In light of pancreatic head prominence and mild elevation in liver function tests, the patient was referred to our center for an endoscopic ultrasound (EUS). The EUS did not show any pancreatic mass with normal common bile duct and pancreatic duct. A transthoracic echocardiogram disclosed normal left ventricular systolic function with no valvular or pericardial structural abnormalities. Given the negative abdominal imaging including EUS, CT and MRI, negative viral serology and normal echocardiogram, and symptom complex consistent with uncontrolled hypothyroidism, we attributed the new onset ascites in our patient to hypothyroidism. Biochemical abnormalities including macrocytic anemia, abnormalities in lipid panel and elevation of CK, ALT and AST (secondary to thyroid myopathy) in our patient were further clues that pointed towards uncontrolled hypothyroidism. She was started on desiccated thyroid since she refused to take levothyroxine. She was counseled about the need for compliance. On a follow-up visit at four weeks, there was no clinical evidence of ascites and the TSH was decreasing (56.31 units/ml).

## Discussion

The pathophysiology of hypothyroidism-induced ascites is still not fully known. There are two main theories: Parving, et al. report that low levels of circulating thyroid hormones cause increase in capillary permeability leading to extravasation of plasma proteins into the extravascular compartment [1, 3, 6-7]. Therefore, analysis of ascitic fluid from patients with this condition shows exudative ascites with high protein content (>2.5 g/dl). A second hypothesis involves a direct hygroscopic effect caused by hyaluronic acid accumulation in the skin with resulting edema. Hyaluronic acid is only found in small quantities in patients with hypothyroidism-induced ascites and cannot be held entirely responsible for exerting the required hygroscopic effect. However, it can interact with albumin forming hyaluronic acid-albumin complexes that prevent lymphatic drainage of extravasated albumin [4-5]. Another theory involves diminished free water clearance due to excess antidiuretic hormone (ADH) production [2, 8]. Recent studies showed that low nitric oxide levels and high vascular endothelial growth factor (VEGF) levels can lead to an increase in capillary permeability causing extravasation of plasma proteins. A decrease in nitric oxide levels causes endothelial dysfunction due to oxidative stress, resulting in activation of inflammatory cells that release substances that increase capillary permeability. An increase in VEGF also leads to increased extravasation of plasma proteins because of abnormal capillary permeability. Levels of VEGF have been reported to be high in hypothyroid patients with a decline in level to normal values with thyroid replacement therapy [9].

Ascitic fluid analysis is the key in guiding the course of investigations needed to establish the diagnosis. A prominent feature of ascitic fluid in hypothyroidism is its elevated protein content but SAAG value varies and can be low or high. A high serum-ascites albumin gradient (SAAG; >1.1 g/dl) suggests that the ascitic fluid is caused by portal hypertension with a 97% diagnostic accuracy [3]. A low SAAG (<1.1 g/dl) suggests that the ascitic fluid is not due to portal hypertension. The common causes of ascites are listed in Table 1.

**Table 1:** Causes of ascites

Table 1: CAUSES OF ASCITES	
High SAAG (≥ 1.1 g/dl)	Low SAAG (< 1.1 g/dl)
Cirrhosis	Peritoneal malignancies
Congestive heart failure	Tuberculous peritonitis
Alcoholic hepatitis	Pyogenic peritonitis
Constrictive pericarditis	Pancreatic ascites
Hepatic metastases	Nephrotic syndrome
Budd-Chiari malformation	Serositis in connective tissue diseases

Our patient did not have clinical features or transthoracic echocardiogram (TTE) findings of congestive heart failure (CHF) or constrictive pericarditis. She did not have a history or clinical findings suggestive of tuberculosis, pancreatitis, nephrotic syndrome or Budd-Chiari malformation. Ascitic fluid cultures were negative for bacterial or tuberculous infections and also showed negative cytology. The abdominal ultrasound showed normal liver parenchyma and the CT scan did not reveal any abdominal masses. In the absence of other causes of ascites and in the presence of long duration of inadequately treated hypothyroidism, myxedema ascites was established as the cause of the patient’s ascites.

Myxedema ascites was first described by Kocher in 1883 [8] and the first case report of ascites associated with hypothyroidism was written by Paddock in 1950 [7-8]. Since then, many case reports have been published. The common features of this condition include female predominance, high protein content of ascitic fluid (>2.5 g/dl), high cholesterol level, long-standing history of hypothyroidism and, most importantly, resolution of ascites with thyroid replacement therapy [4-5, 8]. A review of medical literature showed 51 reported cases of myxedema ascites so far. Ascitic fluid analysis in patients with myxedema ascites consistently shows elevated ascitic protein level (>2.5 g/dl) with a mean of 3.9 g/dl and low [2, 4-5] to elevated [1, 3, 8] SAAG with a mean of 1.5 g/dl and a range of 0.8–2.3 g/dl. WBC counts in ascitic fluid can be low or high, consisting predominantly of lymphocytes [1, 4]. The characteristics of ascitic fluid analysis from this review of literature as well as our patient's ascitic fluid characteristics are shown in Table 2.

**Table 2:** Characteristics of ascitic fluid analysis from review of literature of 51 cases with myxedema ascites and our patient's data

CHARACTERISTICS OF ASCITIC FLUID ANALYSIS FROM REVIEW OF LITERATURE OF 51 CASES WITH MYXEDEMA ASCITES AND OUR PATIENT'S DATA				
	Number of patients	Mean	Ranges	Patient data
Ascitic protein (g/dl)	49	3.9	1.8-5.1	3.9
SAAG (g/dl)	11	1.5	0.8-2.3	1.0
Ascitic WBC count (per µL)	48	60	10-400	497

Certain biochemical abnormalities can point towards the diagnosis of hypothyroidism. These include macrocytic anemia and high creatine kinase and cholesterol levels. Patients with autoimmune hypothyroidism may have concomitant vitamin B12 deficiency caused by pernicious anemia. Hypothyroidism can also cause decreased bone marrow activity and decreased erythropoietin secretion resulting in anemia [10]. The pathogenesis of thyroid myopathy is still not well understood. Isolated serum CK elevation is the most common abnormality. Other clinical manifestations of thyroid myopathy include diffuse myalgias, diffuse muscular hypertrophy associated with weakness and painful muscle cramps (Hoffman's syndrome), slowly progressive symmetric proximal muscle weakness and rhabdomyolysis. The primary mechanism for hypercholesterolemia in hypothyroidism is accumulation of LDL cholesterol due to impairment in lipid clearance caused by a reduction in the number of cell surface receptors for LDL, resulting in decreased catabolism of LDL. A decrease in LDL receptor activity has also been described as a contributing factor. Reduced lipoprotein lipase activity is responsible for hypertriglyceridemia in hypothyroidism.

Our patient's initial laboratory tests showed macrocytic anemia with normal B12 and folate levels, elevated serum CK and AST levels and abnormalities in lipid panel. Physicians should be aware of these minor biochemical abnormalities and should include hypothyroidism as the differential diagnosis in the presence of these abnormalities. These biochemical abnormalities have been reported to normalize after the initiation of thyroid replacement therapy [10].

Myxedema ascites also responds well to thyroid replacement therapy and is completely reversible [4-6]. There is no role for diuretics in this case.

## Conclusions

Severe uncontrolled hypothyroidism is a reversible cause of ascites. Our case illustrates that the diagnosis of myxedema ascites should be kept in mind in patients with uncontrolled hypothyroidism who present with ascites, particularly in patients with high ascitic fluid protein content. It also signifies the need for testing TSH level in addition to other investigations, as part of the workup for ascites of unknown etiology. Physicians should also be aware of biochemical abnormalities that can occur in hypothyroidism such as macrocytic anemia, elevated creatine kinase and abnormal lipid panel and should include hypothyroidism as the differential diagnosis in the presence of these abnormalities.

## References

[1] Philips CA, Sinha U, Chattopadhyay P (2010). Isolated ascites in hypothyroidism: medical and ethical issues. J Indian Med Assoc.

[2] Kabir A, Islam S, Bose A (2015). A male person of 55 years with hypothyroidism, ascites and heart failure. Mymensingh Med J.

[3] Kimura R, Imaeda K, Mizuno T (2007). Severe ascites with hypothyroidism and elevated CA125 concentration: a case report. Endocr J.

[4] Ji JS, Chae HS, Cho YS (2006). Myxedema ascites: case report and literature review. J Korean Med Sci.

[5] Khalil RB, Rassi PE, Chammas N (2013). Myxedema ascites with high CA- 125: case and a review of literature. World J Hepatol.

[6] Malik R, Hodgson H (2002). The relationship between the thyroid gland and the liver. QJM.

[7] Ipadeola A, Nkwocha GC, Adeleye JO (2013). Subclinical hypothyroidism unmasked by preeclampsia and ascites. Indian J Endocrinol Metab.

[8] Subramanian V, Yaturu S (2007). Symptomatic ascites in a patient with hypothyroidism of short duration. Am J Med Sci.

[9] Srinivasan RE, Estes S, Panda M (2015). A rare complication of hypothyroidism: myxedema ascites. Tenn Med.

[10] Marzuillo P, Grandone A, Perrotta S (2016). Very early onset of autoimmune thyroiditis in a toddler with severe hypothyroidism presentation: a case report. Ital J Pediatr.

